# Medical Association of Georgia

**Published:** 1878-05

**Authors:** 


### Editorial.

---

Medical Association of Georgia.—The annual meeting of this Association convened in the Senate Chamber, on Wednesday 17th, and opened with prayer by Rev. J. H. Martin. The address of welcome was delivered by Dr. J. M. Johnson, and responded to by Dr. W. M. Charters, of Savannah.

Dr. Battey, the retiring President, introduced the President-elect, Dr. Wm. O'Daniel, who proceeded to pronounce his inaugural address, touching on various topics, particularly that of rudimentary preparation of medical students and the State Board of Health.

Report of Committee on Annual Assessment of members, which was adopted, required the payment of two dollars by each member in attendance and one dollar from those not present, and that those in arrears shall be discharged from such liability by payment to the treasurer of the said sum of two dollars.

Dr. Love, from the Committee on State Board of Health, reported that circumstances made it prudent to defer application to the last Constitutional Convention.

New members were admitted to the number of about twenty-five, and the whole number in attendance was more than one hundred. The meeting was larger than usual, and had before them more papers on various medical subjects than were ever before presented to the Association at one session. The three days occupied did not afford time to have them all read, to say nothing of the discussion that should necessarily follow the reading. The publication of the Transactions, which is expected to contain these, will certainly be much larger than usual, and will be found to contain interesting and valuable reports.

It was observed that a large proportion of the papers were voluntary communications, the Sections responding poorly, and in some Districts no report at all was received from any of the three sections.

Third District—For the Section on Practice, Dr. Hawkins reported two cases.

Fourth District—For the Section on Practice, Dr. G. J. Grimes read a paper on Tubercular Meningitis. For the Section on Gynæcology, Dr. A. W. Griggs reported four cases dysporeunia; one case antiflexion of the uterus, of five years standing, treated successfully by electricity; one case lateral flexion; two cases ovaritis; two cases lacerated perineum; one case occlusion of the vulva; one case occlusion of the vagina; two cases placenta prævia; one case eclampsia; four cases cancroid; one case hæmatocile; one case of rupture of the uterus where the child was delivered without the aid of forceps.

Fifth District—For the Section on Gynæcology, Dr. W. A. Love reported a case of menstrual derangement resulting from displacement and mechanical obstruction.

Sixth District—For the Section on Surgery Dr. I. L. Harris reported a case of fistula in ano, caused by a chicken bone.

Eighth District—For the Section on Surgery, Dr. A. Mathis reported a case of abdominal dropsy, tapped ninety times, and large quantities of water discharged at each tapping.

Voluntary Papers.—Dr. W. A. Love, on the diagnostic value of the tongue and soft palate; Dr. J. C. LeHardy, on yellow fever; Dr. J. B. Baird, on pathology and theraputics of neuralgia; Dr. Henry Gaither, on puerperal eclampsia; Dr. A. W. Calhoun, one hundred and five operations for strabismus; Dr. W. F. Westmoreland, on congenital phimosis as the cause of nervous disorders; Dr. C. B. Leitner, on application of tar bandages; Dr. Ch. Rauschenberg, on blood letting; Dr. S. H. Stout, on non-syphilitic psoriasis; Dr. T. S. Powell, the true physician; Dr. V. H. Taliaferro, on diseases of the uterus and how to prevent pregnancy; Dr. W. A. Love, on saccharated medicines; Dr. W. T. Goldsmith, on cornstalk pith for uterine tents; Dr. A. W. Griggs reported two cases of tubercular meningitis in support of Dr. Grimes' paper on that subject. Dr. S. H. Stout alluded to the value of veratria ointment as a local application in neuralgia. Dr. A. Means made some remarks on electricity. Dr. W. G. Drake read a paper giving the report of a case in which the entire womb was removed by sloughing. Dr. J. B. Roberts, a case of obstinate hiccough. Dr. J. G. Hopkins, a remarkable case of gunshot wound of the spine.

A motion to create an additional Section in each Congressional District, on "Ophthalmology and Otology," after considerable discussion, was lost.

The annual oration was delivered at noon of the second days' session by Dr. W. R. Burgess, of Macon. Theme: "Hasty, unwise, and unfortunate medical literature." It was well received, and the thanks of the Association was tendered the speaker.

The following officers were elected for the ensuing year:

President—J. T. Johnson; 1st Vice-President—W. F. Holt; 2d Vice-President—T. H. Kenan; Secretary—J. B. Baird; Treasurer—W. R. Burgess; Orator—E. H. Richardson.

On the Sections:

First District.—Practice—J. G. Thomas, Wm. Duncan John D. Martin. Surgery—R. P. Myers, G. A. Stone and J. W. Norton. Gynæcology—J. B. Read, J. C. LeHardy and J. P. S. Houston.

Second District.—Practice—W. M. Bruce, P. L. Hilsman and W. W. Twitty. Surgery—B. R. Doster, W. A.

Strother and T. S. Hopkins. Gynæcology—W. B. Tackett, E. W. Alfriend and T. A. Chappell.

Third District.—Practice—A. R. Taylor, A. W. Reese and S. B. Hawkins. Surgery—F. M. Jordan, G. F. Cooper, and A. A. Smith. Gynæcology—T. F. Walker, J. W. Tucker and J. B. Hinkle.

Fourth District.—Practice—A. W. Griggs, D. W. Johnston and P. M. Tidwell. Surgery—G. J. Grimes, W. W. Fitts and J. T. Slaughter. Gynæcology—J. W. Griggs, F. A. Standford and F. L. Wisdom.

Fifth District.—Practice—J. B. Baird, Paul Faver and J. G. Westmoreland. Surgery—A. W. Calhoun, T. L. Lallerstedt and J. A. McKown. Gynæcology—J. P. Logan, K. P. Moore and T. M. Darnell.

Sixth District.—Practice—W. F. Holt, Henry Gaither and H.V. Johnson. Surgery—I. L. Harris, J. B. Hendricks and S. L. Richardson. Gynæcology—W. O'Daniel, W. R. Burgess and J. E. Blackshear.

Seventh District.—Practice—A. S. Fowler, J. B. S. Holmes and E. H. Richardson. Surgery—R. F. Wright, W. S. Kendrick and W. B. Wells. Gynæcology—R. Battey, C. P. Gordon and F. R. Calhoun.

Eighth District.—Practice—W. H. Doughty, W. W. Battey and W. H. Foster. Surgery—A. S. Campbell, D. Ford and E. A. Dugas. Gynæcology—H. F. Campbell, J. S. Coleman and R. C. Eve.

Ninth District.—Practice—W. T. Hollingsworth, I. H. Goss and J. S. Simmons. Surgery—L. G. Hardman, A. A. Bell and R. M. Smith. Gynæcology—C. W. Long, J.W. Bailey and G. F. Wirsen.

Rome was selected as the next place of meeting.

